# Current Advances in Palliative & Hospice Care: Problems and Needs of Relatives and Family Caregivers During Palliative and Hospice Care—An Overview of Current Literature

**DOI:** 10.3390/medsci7030043

**Published:** 2019-03-12

**Authors:** Karin Oechsle

**Affiliations:** Palliative Care Unit, Department of Oncology, Hematology and BMT, University Medical Center Hamburg-Eppendorf, Martinistr. 52, 20246 Hamburg, Germany; kaoechsl@uke.de; Tel.: +49-40-7410-58774; Fax: +49-40-7410-58841

**Keywords:** palliative care, relatives, family caregiver, needs, quality of life, hospice care

## Abstract

Palliative and hospice care aims to improve quality of life of patients’ relatives, but still little is known about their specific problems and needs. We present a comprehensive literature update. Narrative review to present an expert overview of peer-reviewed, English-written original research publications and reviews on psychosocial and existential problems, supportive needs as well as interventions for relatives during the patients’ disease trajectory published between January 2017 and November 2018. A total of 64 publications were included. Relatives report high rates of psychological and existential distress, burden and psychological morbidity during the total disease trajectory of the patient. In addition, relatives report an alarmingly high number of unmet needs with information being the central issue. Relatives’ problems and needs are part of complex systems influenced by various socio-demographic factors and patient–relatives-interactions and dependency between different psychological phenomena. First support interventions for relatives during disease trajectory have proven feasible and secondary data from randomized studies suggest beneficial effects of providing early palliative care also for relatives. Relatives should be addressed to a still larger extent in the daily practice of palliative and hospice care, thus further research to reveal more detailed systematic information is needed to improve relatives’ psychological burden and quality of life.

## 1. Introduction

Relatives often support or care for patients with life-limiting illness, thus represent important reference persons for them. They have relevant impact on the patient’s well-being, care situation, and quality of life, but at the same time, they are also affected by the patient’s disease in terms of their own specific burden and needs [1,2]. A wide spectrum of psychosocial, physical, and economic factors contribute to the relatives’ burden during the trajectory of the patient’s disease from diagnosis until death and during bereavement [1,2]. Nearing the patient’s death, the relatives’ psychosocial burden can even exceed the patient’s burden [3]. Palliative care aims to improve quality of life not only of the ill persons, but also that of their families and friends [4]. However, significantly less is known about the relatives’ needs and concerns compared to the patients’ to date. Within the last years, an increasing number of studies focus on problems and needs of relatives and family caregivers during palliative and hospice care as well as during bereavement [5,6,7,8]. In addition, initial studies have evaluated supportive measures for relatives at different times of disease trajectory [3]. This publication aims to provide an update of the literature. Recent international research was reviewed to obtain an improved understanding of relatives’ problems and needs during palliative and hospice care for patients suffering from life-limiting illness.

## 2. Materials and Methods

### Methodological Approach

This narrative review includes all relevant peer-reviewed publications on psychological morbidity and burden, quality of life, existential distress, preparedness/self-efficacy, supportive needs as well as on supportive interventions and the role of specialist palliative and hospice care in patients with advanced incurable diseases during disease trajectory published within the last two years. Studies focusing on relatives’ problems and needs during bereavement were excluded to avoid excessiveness due to very heterogeneous aspects. In this literature overview, the definition of relatives also included friends and family or informal caregivers of patients. All types of incurable and/or advanced diseases and all kinds of specific structures of palliative or hospice care were included.

This narrative review did not have a predefined protocol-basis, with the aim to provide a broad overview of current topic-related research. Thus, the adopted search method involved subjective selection and was based on a search from PubMed only. The PubMed search was performed by the author. This review aims to give an update of the literature published between January 2017 and November 2018. The review was limited to original studies and reviews written in English language, but electronic publication (“Epub“)-manuscripts were also included.

The following Medical Subject Headings (MESH) and keywords were used: [relatives], [family caregivers], [informal caregivers], [families], [psychosocial], [existential], [psychological], [needs], [supportive], [quality of life], [advanced], [incurable], [palliative], [terminal], [hospice]. All combinations of search terms describing the cohort “relatives”, potential problems and needs as well as terms describing the specific care situation have been used. The search strings were accessed 16th November 2018. The data extraction included a description of research results and interpretation based on the author’s subjective intention.

## 3. Results

### 3.1. Search Results

Applying the described strategy, a total of 88 full text articles were reviewed. Of those, 24 publications focusing on the bereavement phase only were excluded.

Finally, a total of 64 publications were included with 62 articles being original research (36 quantitative, 16 qualitative, and 10 mixed-methods approach) and 2 being reviews. These articles will be described regarding the main results as well as target groups, patient’s disease situation, and care setting. Appendix A presents an overview on journals of which articles were included in this review

This narrative review does not elaborate on the various different measurements in detail for each study to avoid further confusion.

### 3.2. Basic Characteristics of the Included Studies

#### 3.2.1. Original Research

Regarding the year of publication, 36 out of 62 articles were published in 2017 and 28 in 2018. Countries where the respective studies were conducted were the USA/Canada (*n* = 23) [9,10,11,12,13,14,15,16,17,18,19,20,21,22,23,24,25,26,27,28,29,30,31], Scandinavia (*n* = 7) [32,33,34,35,36,37,38], UK (*n* = 4) [39,40,41,42], Germany (*n* = 4) [43,44,45,46], Southern Europe (Italy, Portugal, Spain; *n* = 8) [47,48,49,50,51,52,53,54], East Asia (Japan, China, Korea, Taiwan; *n* = 4) [55,56,57,58,59,60], Australia/New Zealand (*n* = 4) [61,62,63,64], other (*n* = 4) [65,66,67,68], and multinational (*n* = 2) [69,70].

Forty-seven studies reported on relatives of advanced cancer patient’s [9,10,11,13,15,16,17,18,19,20,21,22,23,24,26,27,28,29,30,31,32,34,36,37,38,39,42,43,44,46,47,48,49,50,51,52,53,55,56,57,58,59,60,61,65,66,67] and 15 studies included relatives of mixed patient samples [12,14,25,33,35,40,41,45,54,62,63,64,68,69,70]. The environment in which the patient received palliative or hospice care were home care in 18 studies [10,14,25,26,27,30,33,35,36,37,38,41,42,44,45,54,58,62], specialist inpatient palliative care in four studies [34,43,55,67], inpatient hospice care in 3 studies [13,49,60], mixed settings in 25 studies [9,11,13,19,20,21,22,23,24,28,29,31,32,39,40,47,51,53,56,60,61,63,65,67,70], and 12 other settings/not described [15,16,17,18,46,49,50,52,57,66,68,69]. In 45 studies, the sample only consisted of relatives reporting on their own situation [9,10,11,12,13,14,15,16,18,19,21,22,23,24,28,29,30,31,32,34,37,38,39,41,43,44,45,47,48,49,50,51,54,55,56,57,58,59,61,62,63,64,65,67,70], while the remaining studies collected data from mixed populations (e.g., relatives, patients, and health care providers) or health care providers. Out of 42 publications reporting the average age of included relatives, the overall mean age was approximately 57 years.

#### 3.2.2. Reviews

The review included two review articles. The first was a scoping review focusing on relatives of dementia patients at end of life including 22 studies published between 2000 and 2016 [71]. The second was a systematic review focusing on unmet needs of relatives of cancer patients [72]. This review included 50 studies from initiation of the used databases until 2017.

### 3.3. Relatives’ Psychological Morbidity and Burden

#### 3.3.1. Psychosocial Distress

High levels of psychosocial distress have repeatedly been reported in previous literature and three new studies strengthened these findings, reporting high or significant levels of psychosocial distress in 66–96% of family caregivers in different palliative care settings [43,47,65]. Ullrich et al. used an adapted problem list of the Distress Thermometer [73] in their pilot study to investigate distress-causing problems, with sadness, sorrows, and exhaustion being the most relevant (80–83%) [43]. In addition to previously reported factors, higher distress levels seem to be associated with a higher number of unmet needs [61] and dissatisfaction with patient care [73].

#### 3.3.2. Anxiety and Depressive Symptoms

At admission to a palliative care ward, moderate to severe anxiety and depression were prevalent in 43% and 41% of family caregivers of advanced cancer patients, respectively [43], which is in line with two larger new studies: Dionne-Odom et al. presented rates of 23% for high depression and 34% for borderline or high anxiety in 294 family caregivers of advanced cancer patients [9] and Goetze et al. reported high anxiety levels of 32% and high depression levels of 29.2% in family caregivers of palliative cancer patients during home care [43]. Using other measurements, Areia et al. observed a high risk of anxiety in 72% and a high risk of depression in 69% in 112 family caregivers of patients with terminal cancer. In addition, 51% of family caregivers reported high levels of somatization [47]. In a randomized controlled study, the group of Jennifer Temel evaluated psychological morbidity in patients and family caregiver dyads in early palliative care for patients with newly diagnosed advanced lung or non-colorectal gastrointestinal cancer. They showed that patients reported more depressive symptoms and caregivers reported more anxiety symptoms. Dyads’ anxiety and depression symptoms were both positively associated [10]. Other parameters associated with family caregivers’ psychological morbidity were younger age, female gender, relationship to the patient, the caregiving role played, satisfaction with health care professionals, shorter nighttime sleep, less physical activity, family functioning, and low engagement in self-care practices [9,11,39,47]. Death anxiety was associated with dysfunctional attitudes in 173 caregivers of advanced lung cancer patients [56].

#### 3.3.3. Overall (Psychological) Burden

Overall psychological burden in relatives seems to depend on anxiety, depression, patient age, gender, and changes in meaning [12,45,48]. In a qualitative study, Williams et al. demonstrated that female family caregivers’ burden can also be caused by the adherence to stereotypical gender norms that affect women as primary caregivers [62]. In patients with newly diagnosed lung cancer, the self-efficacy of family caregivers was observed to be the strongest factor for their psychological burden during the patients’ total disease trajectory [57]. Perceived competence, resilience, and positive aspects of caring represented the main predictors of burden in 50 informal caregivers of patients with advanced cancer [65].

#### 3.3.4. Role of Care Setting and Utilization of Assisting Services

In family caregivers of patients receiving palliative or hospice home care, the psychological burden seems to be associated with managing the patients’ psychological or psychiatric symptoms mediated by caregivers’ use of escape-avoidance coping responses, their own poor health status, and being the spouse of the patient [12,58]. Kobayakawa et al. also reported that 11% of caregivers had consulted psychiatrists or psychologists to become able to manage the patients’ symptoms, while another 11% expressed the wish/plan to do so [58]. A large analysis of 373 caregivers of cancer patients observed that providing assistance managing medical care was associated with increased emotional and psychological burden, while assistance with non-medical issues increased psychological burden and impaired the relationship with the patients. Providing direct patient care activities also increased the burden but improved relationships with patients, while use of support showed mixed associations with the burden [13].

#### 3.3.5. Role of Relatives’ Satisfaction with Care

Family satisfaction with end-of-life care also seems to have a relevant impact on caregiver burden: in family caregivers of patients receiving palliative outpatient care dissatisfaction was associated with caregiver distress, loss of control, personal time, social engagement with others, being angry with the patient, feeling that the patient wants more help than needed, and the wish to leave patient care to someone else. Dissatisfaction was mainly caused by insufficient information regarding the patients’ prognosis, inadequate family conferences or inadequate way of involvement in care decisions [55].

### 3.4. Pre-Loss Grief, Confidence, Preparedness, Self-Efficacy, Hope, and Spirituality

#### 3.4.1. Pre-Loss Grief

A large study evaluating almost 3000 family caregivers during end-of-life care reported severe pre-loss grief symptoms in 15%. Depressive symptoms, high caregiver burden, low preparedness for death, low level of communication about dying, and “too much” prognostic information were associated with pre-loss grief symptoms in these family caregivers [32]. Another study reported a high risk of complicated anticipatory grief in 26% of family caregivers of patients with terminal cancer [47].

#### 3.4.2. Relatives’ Confidence and Sense of Security, Preparedness and Self-Efficacy

Soroka et al. qualitatively analyzed family caregivers’ confidence during hospice home care and observed four “storylines” contextualizing caregivers’ confidence which were: “values/relationships”, “stories of terminal illness”, “needs”, and “support” [14]. Lower caregiver decision-making confidence was associated with less engagement in spiritual growth self-care, more use of avoidant coping, low emotional social support, and younger care recipient age in a large evaluation in 294 family caregivers of advanced cancer patients with the strongest associations for low spiritual growth self-care and high use of avoidant coping [15]. The same working group had observed that lower decision-making self-efficacy and preparedness were more frequent in advanced cancer patients’ family caregivers with lower health responsibility, spiritual growth, interpersonal relation, and stress management scores [9]. Control over decision making for patient care and communication were also reported to have an impact on family conflicts during end-of-life care. At least one family conflict was reported by 42% of family members, although absent family members reappearing unexpectedly reduced family conflict [59]. Family members’ sense of security in caregiving also seems to be positively influenced by the availability of attachment figures. Both nonphysical and physical contact with the attachment figures promote a sense of security and facilitate the diversion of attention from the patients’ illnesses to other things in everyday life. This study identified four relevant types of attachment figures: “family and friends, health care practitioners, pets and God” [35]. In a qualitative analysis exploring family caregivers’ preparation for the patient’s death in the context of palliative care, caregivers emphasized “the complexities of trying to prepare while feeling overwhelmed with demands of caregiving throughout an unpredictable illness trajectory”. Cognitive preparedness was feasible for most of them, behavioral preparedness for some, but emotional preparedness remained challenging [63].

#### 3.4.3. Resilience

Two recent studies explored the impact of resilience of relatives. Hwang et al. were able to demonstrate that in 273 family caregivers from hospice and palliative care units, high resilience was associated with their perception of good health, positive social support, and absence of depression [60]. Caregiver resilience also seems to be influenced by cognitive dysfunction in patients with brain metastases, with 77% of caregivers indicating moderate to high resilience. The most frequently used coping strategies were acceptance, planning, positive reinterpretation, and growth [16].

#### 3.4.4. Spiritual Aspects

Relatives’ spiritual concerns have been addressed by four studies. Selman et al. explored spiritual concerns of family caregivers of patients with life-limiting diseases performing focus group interviews in 11 countries all over the world. They could observe four categories: existential (“questioning why them?”; “questioning why me?”; “looking for meaning”), psychological (“guilt”; “stress, feeling overwhelmed”; “questioning ability to cope”; “worry about the future, including role as carer”; “difficulties with acceptance”; “isolation, loneliness of caregiving”; “feeling insufficient, not knowing what to do”), religious (“anger at God/questioning God”), and “social and relational” (“relationship, including change in relationship with the patient”) [69]. In addition, family caregivers reported that spiritual care was insufficient due to its lower prioritization by health care professionals and lacking time [69]. Lai et al. reported that higher levels of intrinsic spirituality were associated with a higher amount of time devoted to caregiving. Intrinsic spirituality also had a protective effect on emotional distress linked to providing assistance in caregivers of terminally ill cancer patients [48]. Spattuzi et al. and Vespa et al. both similarly observed that higher spiritual well-being was associated with better outcomes related to mental health, bodily pain, vitality, social activities, and physical and developmental burden in family caregivers of advanced cancer patients [49,50].

### 3.5. Relatives’ Quality of Life, Physical Health, and Well-Being

#### 3.5.1. Relatives’ Quality of Life

Ullrich et al. reported impaired health-related quality of life in all subscales with the exception of “bodily pain” in family caregivers of advanced cancer patients on a palliative care ward compared to the German standard population [43]. Goetze et al. also observed impaired quality of life compared to the German standard population from the beginning of palliative home care in advanced cancer patients compared to two months after the patient had died. They could not detect changes in quality of life over time comparing these two assessment points [44].

In their large evaluation of 294 family caregivers of advanced cancer patients, Dionne-Odom et al. observed that low engagement in self-care practices is associated with worse family caregiver mental health-related quality of life [9]. As mentioned above, in family caregivers of advanced cancer patients, quality of life (mental health, bodily pain, vitality, social activities) is also affected by their spiritual well-being [49,50].

#### 3.5.2. Patient-Relative-Dyads and Quality of Life

In addition to these factors, related to the relatives themselves, three studies analyzed patient-relative-dyads effects in relation to their quality of life. In a cohort of 275 dyads of patients with newly diagnosed cancer, younger, spousal caregivers, who cared for patients with low emotional well-being, also indicated worse mental health themselves. Further, family caregivers with low educational attainment, who cared for patients with low social well-being, reported worse physical health themselves [17]. Another study focusing on lung cancer patient-caregiver-dyads demonstrated that caregiver quality of life was associated with dependency, patient self-control and achievement. Patient self-control and family caregiver dependency mediate between patient death anxiety and caregiver quality of life [58]. A third study on cancer patient-caregiver-dyads demonstrated that “the use of positive thinking and future/present-hedonistic perspectives” was also associated with higher quality of life. “The use of avoidance” and “past-negative perspective” were related with lower quality of life. When caregivers used social support and experienced openness, the patients’ quality of life was higher. On the other hand, when patients used social support, avoidance strategies and experienced future perspective, the caregivers’ quality of life was lower [68].

#### 3.5.3. Role of Care Setting and Caregiving Role on Quality of Life

Spatuzzi et al. demonstrated that family caregivers of cancer patients receiving hospice care reported significantly lower quality of life scores in the mental component and higher scores in general health and in the physical component than those of patients during active cancer treatment [53]. This might either demonstrate that relatives’ quality of life depends on the patients’ care setting or might again strengthen effects between patients’ hope for future perspective facilitated by active treatment and relatives’ quality of life. A mixed-method study, which explored links between quality of life and self-reported caregiver communication identified four caregiver communication types: “manager”, “carrier”, “partner”, and “lone caregiver”. “Manager” reported the lowest physical quality of life, while “carrier” felt least prepared and “partner” and “lone caregivers” reported lowest psychological distress [20].

#### 3.5.4. Physical Health and Well-Being

Shaffer et al. observed even slightly better physical health in 664 family caregivers of patients with newly diagnosed lung cancer compared to the USA national population, however physical heath significantly declined over the following six years [17].

In another large study by Shaffer et al. evaluating physical health in family caregivers from newly diagnosed lung cancer over the following 6 years, all socio-demographic factors (age, gender, education, income, relationship to the patient, employment status), patient cancer severity, and caregiving stress were associated with the family caregivers’ initial physical health. Higher depressive symptoms were the only significant predictor of family caregivers’ more rapidly decreasing physical health, although they were not associated with their initial physical health [19].

#### 3.5.5. Patient-Relative-Dyads and Physical Health

In a study analyzing 33 head and neck cancer patient-caregiver-dyads, family caregivers’ physical well-being was higher in family caregivers of older age, with more than two comorbidities, at least seven hour of sleep per day, physical activity at least three times per week as well as swallowing and speech dysfunction of the patient, which was partly similar for anxiety and depression as mentioned above [11]. A scoping literature review reporting on relatives of patients with dementia during the end of life reported that their relatives’ well-being was influenced by their experience of relationships with the patients, family and friends, and health care professionals as well as the specific context of caring for someone with dementia [71].

### 3.6. Relatives’ Supportive Needs

#### 3.6.1. Attempts to Categorize and Prioritize (Unmet) Needs

In 2018, a large systematic review analyzed current literature on unmet care needs of advanced cancer patients’ informal caregivers. First, the authors had to state that current data are characterized by significant heterogeneity regarding study contexts, assessment methods, measurement instruments, classifications, and report methods. Further, the majority of studies evaluated unmet needs in cross-sectional designs. However, they worked out that “information” was the central theme in the context of unmet needs. The two main information categories were either “illness or treatment information” (26–100%), or “care-related information” (21–100%) [72].

Several new studies tried to further elucidate the complex construct of relatives’ needs. A qualitative interview study revealed three main categories of needs in family caregivers of end-of-life cancer patients, which were “social needs” (“support for care, effective communication and financial support”), “cognitive needs” (“educational support and support in decision-making”, and “psychological needs” (“support for psychological trauma, preparation to confront the reality of the death of a loved one, and support for mourning”) [67]. The most frequent needs of family caregivers of neuro-oncological patients during hospitalization and after discharge were of material and informative nature. Specific needs tend to decrease over time; in particular “need for knowledge about the disease” and “information/communication needs [52]. Weißflog et al. analyzed the specific needs of patients with hematological cancer and their partners and could demonstrate that their supportive care needs were lower in the dimensions “health system/information” and “physical problems/daily living” compared to an advanced cancer patients’ validation sample. Partners’ supportive care needs were higher in cases perceiving the patient’s negative dyadic coping, while socio-demographic and illness-related factors were only partially associated with the partners’ supportive care needs [46].

#### 3.6.2. Specification of Relatives’ Needs Related to Transitions

Three studies present further details on relatives’ needs regarding the patients’ disease and care transitions [20,21,68]. Interviews with older caregivers of cancer patients one and two weeks after the patient’s discharge from hospital addressed three main themes which were “connection of caregiver and patient wellness”, “caregivers’ struggle with control issues” and “communication with health professionals” [20]. Maintaining normality turned out to be the central need during transitions in families of patients receiving palliative care in a large qualitative meta-analysis. The authors also observed that transitions are often experienced unconsciously until occurrence of crisis, when active responses are needed which could encourage families to develop coping strategies. With progressive disease, family caregivers have to repeatedly reprioritize and balance their caregiving roles [68]. Norton et al. qualitatively analyzed relatives’ needs resulting from different transition patterns and identified three different types of transitions experienced by family caregivers of cancer patients when stopping chemotherapy and transiting to end-of-life care. First, “we pretty much knew” (explicit discussions about end-of-life care with shared understanding about prognosis and seamless transitions), second “beating the odds” (explicit discussions about disease-directed treatment and end-of-life care, but no shared understanding about prognosis and often chaotic transitions) and finally, “left to die” (no end-of-life discussion with transitions resulting in crisis) [21].

#### 3.6.3. Quantification of Needs

Regarding the number of needs of relatives, Halkett et al. demonstrated that the mean numbers of moderate to high needs did not change, but specific needs changed over time in family caregivers of glioma patients. The most frequently reported needs included the impact of caring on their working life or usual activities, decision making in the context of uncertainty, reducing the patient’s stress, and understanding the patient’s experience [61]. Family caregivers of cancer patients scored a mean number of 16 out of 20 needs as very or extremely important, however, 20% of these important needs were unmet in at least half of the family caregivers at first admission to a palliative care ward. These unmet needs were: “feel there is hope”, “know when to expect symptoms to occur”, “have somebody be concerned with my health”, and “be told about people who could help with problems” [43].

### 3.7. Supporting Relatives During Disease Trajectory

#### 3.7.1. Training Relatives for Caregiving

In a large USA study with more than 600 informal caregivers of cancer patients, 59% reported that they did not receive training for the care provided although they had to perform “some type of medical/nursing task”. Lacking access to training was associated with higher levels of psychosocial burden, which was partially mediated by confidence [22]. In another study, informal caregivers of colorectal cancer patients reported inadequate training for fatigue in 48%, for pain in 38%, for bowel control in 38%, for medication administration in 26%, and for other symptoms in 40%. Self-efficacy in caregivers was significantly associated with training adequately supporting the impact of cancer symptom management training for relatives [23]. Ewing et al. suggested that current barriers impeding appropriate support for relatives at hospital discharge might be an organizational focus on patients’ needs, “unrealistic expectations” on end-of-life caregiving at home perceived by health care professionals and lacking awareness of patients’ end-of-life situation [40].

#### 3.7.2. Interest in and Use of Support Services

Regarding relatives’ use of support services, Dionne-Odom reported that only 32% of almost 300 family caregivers of advanced cancer patients accessed support services, while further 28% indicated to be “mostly” or “extremely” interested. Accessing support services or being interested was more frequent in caregivers with depressive or anxiety symptoms or low preparedness. Interest was also associated with belonging to a minority, shorter caregiving durations, and higher burdens [24].

#### 3.7.3. Preferences of Being Involved in and Educated about Patient Care

A large Italian study investigated the desired involvement in patient education and empowerment activities of cancer patients and their caregivers. Almost half of them, mainly caregivers, indicated that they would like to be involved. The preferred educational activities were “classes on cancer-related topics with health care professionals” and “cancer information service” on a face-to-face modality [53]. Support for home hospice family caregivers might be grouped in four categories including “skilled communication”, “building authentic relationships”, “expert teaching”, and “teamwork”. From the perspective of family caregivers, “expert teaching” might be the most important [25]. An analysis of 101 statements of hospice nurses describing caregivers’ pain management challenges revealed that 30% of these statements addressed communication and teamwork issues and 27% caregivers’ medication skills and knowledge. Hospice nurses responded to caregivers’ pain management challenges with a validating statement in 52% and with further information in 42% [26].

#### 3.7.4. Role of Health Care Providers

Røen et al. asked family caregivers “how can health care providers contribute to resilience for family carers of advanced cancer patients?” and four main factors were identified: family caregivers want be seen and known by health care providers (building a personal relationship), palliative care should be available, information and communication about illness, prognosis, and death should be offered, and a good caregiver–patient relation should be facilitated [34].

### 3.8. Interventions to Support Relatives During Disease Trajectory

#### 3.8.1. Supporting Relatives by Development of Support Plans

The Carer Support Needs Assessment Tool (CSNAT) approach aims to expand the focus of discharge planning on caregivers’ support needs, to strengthen the carers’ role in end-of-life care and to prevent patients from hospital readmission due to decompensation of home care, which was deemed feasible by carers and health care professionals [41]. In addition, the CSNAT intervention seems to reduce early grief, improve psychological and physical health as well as the perception that the “place of death was the right” in family carers compared to the control group, and to increase the rate of patients dying at home [41]. Thomsen et al. evaluated a four-step process evaluation of systematic risk and needs assessment for caregivers in specialized palliative care. They were able to include 46% of their family caregivers in the intervention. The first steps, an interdisciplinary risk assessment and documentation of a support plan could be performed in 75%, whilst the final step, a separate medical record according to the intervention blueprint, could be completed in 62% of caregivers. The palliative care staff perceived that this intervention was useful and acceptable, but efficacy data are pending [35].

#### 3.8.2. Supporting Relatives in Symptom Management

Another new intervention, the “Cancer Carers Medicines Management” was developed to support carers with the management of pain medication in advanced cancer patients during end-of-life care. This approach was also feasible and acceptable for participating carers and nurses, however efficacy data have not yet been presented. In addition, the authors highlight the typical recruitment problems in studies including relatives or carers, when eligible participants remain fewer than expected and the so called “gate-keeping effect” occurs, meaning that health care professionals rate many potential participants as unsuitable for participation to prevent them from additional burden [42].

A telephone symptom management intervention delivered simultaneously to lung cancer patients and their caregivers aimed to examine associations of coping skill practice to symptom change. In family caregivers, greater practice of guided imagery was associated with less psychological distress, but many negative unexpected effects on patients’ physical and psychological well-being suggest that different intervention might be beneficial for patients and their family caregivers [27].

#### 3.8.3. Supporting Cooperation and Communication within Families

Finally, Forbat et al. evaluated the effects of family meetings during specialist inpatient palliative care and could demonstrate that families perceived more empathy by staff afterwards. However, facilitating effects were hampered by pre-existing dynamics in families with longstanding previous communication problems. The authors suggest specific preparation in families who have a history of conflict or complex dynamics and during discharge planning [64].

### 3.9. Impact of Different Concepts of Palliative and Hospice Care

#### 3.9.1. Effects of Integrated Palliative Care

A multinational mixed-methods study indicated that there is some evidence that integrated palliative care initiatives with “well-developed professional care networks and communication systems” might reduce burden in family caregivers by direct (e.g., provision of night shift nurses) and indirect effects (psychological support). However, systematic or institutionalized support structures for family caregivers were not established in any country [70].

#### 3.9.2. Effects of Early Specialist Palliative Care

In a Canadian cluster-randomized study on early palliative care in advanced cancer patients, a significantly improved family caregiver satisfaction with care could be observed in the palliative intervention group compared with controls over three and four months. In contrast, family caregivers’ quality of life did not significantly differ in both groups [28]. An additional qualitative interview study on family caregivers’ quality of life worked out the core category “living in the patient’s world” with the related themes “burden of illness and caregiving”, ”assuming the caregiver role”, “renegotiating relationships”, “confronting mortality”, and “maintaining resilience”. In two themes, there was no consistency between the two groups: caregivers in the palliative care intervention group were more likely to “engage in open discussion about the end of life, balanced hope with realism and had increased confidence from a range of professional supports”, while those in the control group were more likely to engage “in deliberate ignorance about the future, felt uncertain about how they would cope and lacked knowledge of available supports” [29]. A second qualitative study on family members retrospective perspective on providing home care six months to five years after the patient’s death did not reveal any differences in themes between the control and intervention groups [30].

A Danish working group has developed a specialist palliative care and dyadic psychological intervention focusing on patient-caregiver-dyad, psychological distress, existential concerns, interdisciplinary collaboration, and need-accorded psychological interventions (DOMUS trial) which was feasible and acceptable for patients and caregivers [36]. The randomized controlled study demonstrated that this intervention can significantly reduce the increase in caregivers’ anxiety and depressive symptoms during the patients’ disease trajectory at least until two months in bereavement [37]. Further, significant effects on coping strategies in patients and caregivers as well as on stress communication by partner caregivers could be observed [37].

#### 3.9.3. Effects of Hospice Care

Regarding potential effects of hospice care in relatives of patients with life-limiting diseases, a prospective cohort study observed that family caregivers of cancer patients receiving hospice care reported significantly lower quality of life scores in the mental component and higher general and physical health than those of patients during active cancer treatment [38]. As discussed above, this might be caused by hospice care or by the psychological effects of active treatment. A large study asked family members of deceased patients with advanced cancer about their perspectives on the patient’s hospice care experiences. It was demonstrated that families of patients in a hospice context reported more pain for the patient compared with those without hospice care. However, they reported more frequently that the patients had received “just the right amount of pain medicine” (80% vs. 73%), more help with dyspnea (78% vs. 70%), that patients’ wishes on end-of-life care were followed (80% vs. 74%) and that quality of end-of-life care was “excellent” (57% vs. 42%) [31].

#### 3.9.4. Effects of Palliative Home Care Services

Potential effects of a palliative home care service were explored in a Spanish content analysis that evaluated written gratitude documents from bereaved relatives. They could work out three different content categories: “recognition of the care received and the value of particular aspects”, “family recognition of the achievements of the palliative care team”, and “messages of support related to the need of resources provided”. The authors highlight the key role of relational aspects as an indicator for high quality palliative care [54].

## 4. Discussion

This narrative review demonstrates the impressive amount of new research on psychosocial and existential problems, supportive needs, and potential interventions for relatives of patients with life-limiting diseases, with 64 new publications focusing on the period during the patient’s disease trajectory. The review included 62 original research articles (73% cancer-specific) and two reviews, which were published within the last 24 months. The rapidly increasing number of publications on this issue reflects the growing awareness of the importance to not only care for the patient, but to also include their relatives in palliative and hospice care perspective and activities.

The presented studies focus on many different facets of relatives’ problems and needs, reflecting the enormous spectrum of aspects affecting relatives. However, the various different and heterogeneous aspects currently complicate the development of a comprehensive picture of the relatives’ situation. The presented studies are not only related to a broad spectrum of issues, but also used different target groups (relatives, family caregivers, families and friends, informal caregivers, family carer, carer, etc.) and analyzed different care settings in different life-limiting diseases at different stages and with different cultural backgrounds. In the context of those studies, very heterogeneous sets of potential risk factors have been analyzed impeding the comparison of results among each other. Many studies are cross-sectional studies and in the few longitudinal studies, different time points have been analyzed. Furthermore, the use and interpretation of terminology is inconsistent over the various studies, e.g., regarding the terms “burden” and “psychological morbidity”. In addition, some constructs, e.g., “quality of life” were measured and interpreted inconsistently. Finally, many different measurements and instruments were used for quantitative assessments with some of the questionnaires being not validated for respective indications.

Overall, the discussed studies strengthen previous data reporting high rates of psychological and existential distress, psychosocial burden, and psychological morbidity in relatives during the patients’ disease trajectory [1,2,3,4,5,6,7,8]. Some further studies indicated that psychosocial problems and symptoms might decrease over time after the patient’s death [44,74,75], but typical trajectory during the patient’s disease and long-term data on further course after the patient’s death need to be systematically evaluated in future research. Impaired quality of life in relatives of patients with life-limiting illness had consistently been reported in various previous studies [1,3], these new studies strengthen this phenomenon and add further information on influencing factors [43,44].

Relatives report an alarmingly high number of unmet needs with “information” being the central theme [72], thus the development of further information and support strategies for relatives is urgently required. Strategies have to be tailored to relatives’ preferences of how, when, and from whom information and support is needed and considered to be helpful. Relatives’ problems and needs are impacted by many different risk factors, including socio-demographic, care-related, and patient-related factors as well as various interactions between different own psychological phenomena [9,10,11,12,39,44,45,47,48,49,50,56,57]. Future research should also further elucidate these complex interactions and mutual mediations of different psychosocial, existential, socio-demographic, and disease-related factors impacting their situation. Having identified potential risk factors allows improvements of supporting relatives adequately to reduce their burden. Although some risk factors may be non-modifiable (e.g., socio-demographic- or disease-related factors), it is clinically important to increase health care providers’ awareness and enhance early identification of at-risk relatives. On the other hand, knowledge about modifiable risk factors (e.g., care-related factors, other psychological phenomena) may help to develop supportive care measures addressing these factors more adequately in future. First support interventions for relatives have proven feasibility [27,35,41,42,64], but efficacy analysis and further adapted models are still pending. Furthermore, randomized studies suggest beneficial effects of extended early palliative care including also the patients’ relatives, however relatives-related outcomes represented mainly secondary end points in relevant studies [19,28,36,37,38]. Therefore, further palliative care and/or psychosocial interventions focusing on relatives or on patient-relative-dyads are needed.

Obviously, this narrative review has several limitations. Despite the author’s effort to review all relevant topic-related studies from the last two years, possibly some could be missed. The review involves subjective selection bias due to methodological reasons: Search media was restricted to one search engine (PubMed) and the literature search was not pre-defined protocol-based. Further, the language restriction to English also limits the scope of the review. However, the presented narrative review provides a broad overview and update of the literature on the topic of problems and needs of relatives of patients with life-limiting illness from an expert’s perspective.

In conclusion, psychosocial and existential problems and supportive needs of relatives of patients with life-limiting diseases are clinically relevant during the patients’ disease trajectory and should not be underestimated in daily clinical practice of palliative and hospice care. Studies illustrate complex constructs of different problems, needs, and influencing factors, but comprehensive understanding of the relatives’ situation remains limited. On the one hand, further systematic and longitudinal studies are needed to analyze their situation, whilst on the other hand, supportive interventions have to be developed and evaluated in randomized trials to further improve supportive care for relatives in daily palliative care and hospice practice.

## Appendix A

**Overview on journals included in this review:**

- *American Journal of Hospice and Palliative Medicine.*
- *Annals of Behavioral Medicine.*
- *Annals of Oncology.*
- *BMC Palliative Care.*
- *BMJ Supportive & Palliative Care.*
- *British Journal of Cancer.*
- *Cancer.*
- *Death Studies.*
- *European Journal of Cancer Care.*
- *Journal of Clinical Oncology.*
- *Journal of Pain and Symptom Management.*
- *Journal of Palliative Care.*
- *Journal of Psychosomatic Research.*
- *Palliative Medicine.*
- *Palliative & Supportive Care.*
- *Psycho-Oncology.*
- *Quality of Life Research.*
- *Support Care in Cancer.*
